# Detection of Gray Mold Leaf Infections Prior to Visual Symptom Appearance Using a Five-Band Multispectral Sensor

**DOI:** 10.3389/fpls.2019.00628

**Published:** 2019-05-15

**Authors:** Johannes Fahrentrapp, Francesco Ria, Martin Geilhausen, Bernd Panassiti

**Affiliations:** ^1^Institute of Natural Resource Sciences, ZHAW Zurich University of Applied Sciences, Wädenswil, Switzerland; ^2^Independent Researcher, Munich, Germany

**Keywords:** disease imaging, tomato, gray mold, *Solanum lycopersicum*, *Botrytis cinerea*, early disease detection, symptom detection, linear predictive model

## Abstract

Fungal leaf diseases cause economically important damage to crop plants. Protective treatments help producers to secure good quality crops. In contrast, curative treatments based on visually detectable symptoms are often riskier and less effective because diseased crop plants may develop disease symptoms too late for curative treatments. Therefore, early disease detection prior symptom development would allow an earlier, and therefore more effective, curative management of fungal diseases. Using a five-lens multispectral imager, spectral reflectance of green, blue, red, near infrared (NIR, 840 nm), and rededge (RE, 720 nm) was recorded in time-course experiments of detached tomato leaves inoculated with the fungus *Botrytis cinerea* and mock infection solution. Linear regression models demonstrate NIR and RE as the two most informative spectral data sets to differentiate pathogen- and mock-inoculated leaf regions of interest (ROI). Under controlled laboratory conditions, bands collecting NIR and RE irradiance showed a lower reflectance intensity of infected tomato leaf tissue when compared with mock-inoculated leaves. Blue and red channels collected higher intensity values in pathogen- than in mock-inoculated ROIs. The reflectance intensities of the green band were not distinguishable between pathogen- and mock infected ROIs. Predictions of linear regressions indicated that gray mold leaf infections could be identified at the earliest at 9 h post infection (hpi) in the most informative bands NIR and RE. Re-analysis of the imagery taken with NIR and RE band allowed to classify infected tissue.

## Introduction

Agricultural plant production relies on numerous applications of pesticides against an army of pathogenic organisms including virus, bacteria and fungi. Today, both Swiss and European policy aims at a drastic reduction of pesticide applications as well as active compounds such as copper or neonicotinoids (EU Directive 2009/128/EC). A curative and more site-specific treatment of, e.g., single crop plants or distinct infected plots in a field could contribute to limit pesticide diffusion to the environment. To achieve this goal, an early detection of pathogen infection is a basic requirement. However, symptom detection by experts is time consuming and often too late for curative treatments. With imaging technologies site-specific application systems as for example against grape downy mildew could be established (Oberti et al., 2016). Remotely sensed reflectance imaging allowing to identify non-destructively and “on-the-go” diseased plants could become key in optimized application strategies with lower number of applications.

Currently, the common methods to identify fungal leaf diseases are symptom detection by either naked eye observation by experts or via smart phone applications^1^. Additionally, destructive molecular tests like ELISA and latera flow (e.g., Braun-Kiewnick et al., 2011), RT-PCR (e.g., Gachon and Saindrenan, 2004; Suarez et al., 2005; Fahrentrapp et al., 2013), LAMP-PCR (e.g., Pelludat et al., 2009) can be used to identify causal agents. Results obtained with these methods are either too late for curative treatments (symptom detection) or destructive and laborious. However, thermal sensors, multi- and hyperspectral sensors can be used to spot leaf diseases (e.g., Mahlein et al., 2012, 2018). The spectral information then can be used to identify leaf diseases. Leaf reflectance in the visible range (including red, green, and blue), near-infrared (NIR) and short-wave infrared (SWIR) is mainly influenced by pigments, leaf structure and internal scattering, and water and chemical absorption, respectively (Mahlein, 2016). In-between the visible and NIR range, the so called “rededge” (RE) region describes a steep slope in the spectral reflectance of plant material that is often used to build several disease indices (Lowe et al., 2017). Biotrophic and necrotrophic fungal diseases can have rather low and high impact on leaf structure, respectively, and thus also on leaf reflectance. However, as demonstrated in sugar beet, leaf diseases such as *Cercospora* leaf spot (*Cercospora beticola*), powdery mildew (*Erysiphe betae*), and rust (*Uromyces betae*) could be differentiated under laboratory conditions by means of hyperspectral imaging (Mahlein et al., 2010, 2013), and diseases were identified before visible symptoms developed (Rumpf et al., 2010; Lowe et al., 2017). Obstacles to detect disease under field conditions are mainly (1) the resolution of suitable sensors, (2) differing light environments under field conditions, (3) leaf angle to sensor, and (4) shadows of overlapping leaves (Thomas et al., 2018). For instance, an increased view angle correlates with an increased sensitivity peaking at 60° (Oberti et al., 2014). Additionally, sensor costs may be high especially when hyperspectral information is needed (Grieve et al., 2015). Low-cost multispectral sensors equipped with a LED-based narrow band illumination demonstrated comparable results in disease detection as hyperspectral imagery (Grieve et al., 2015). In our study, we used an “off-the-shelf” multispectral camera, the MicaSense^®^ Rededge (Seatle, WA, United States), under laboratory settings to demonstrate its use in early detection of a fungal disease on leaves. The MicaSense^®^ Rededge is a snap-shot camera collecting five distinct bands of less than 40 nm in the red, green, blue, NIR, and “rededge” range on a sensor through five individual lenses. Such sensors are currently available for less than 3500°C. Our proposed methodology (including image processing and data analyses) targets rather service provider and agricultural research institutes than producers.

Gray molds such as *Botrytis cinerea* Pers. (1794) are important plant diseases all over the world. We used *B. cinerea*, being the second most important plant pathogen worldwide (Dean et al., 2012), as example leaf disease in the presented work. *B. cinerea* is a necrotrophic fungus affecting both annual crops (e.g., *Solanum lycopersicum, Fragaria* species) as well as perennials such as *Vitis vinifera* (Hennebert, 1973, Staats et al., 2005, Elad et al., 2016). It is the causal agent of gray mold on leaves and fruits in a large number of plant species (Williamson et al., 2007). Symptoms caused by *B. cinerea* infection become visible in leaves approximately 24–48 h post infection (hpi) (Asplen et al., 2015). In tomato (*S. lycopersicum* L.), both leaves and fruits are attacked by *B. cinerea*. Detection of leaf infections are of high importance since they can cause severe plant damage, lead to less and low-quality fruits, and increase spore density as inoculum for fruit infections. In Switzerland, 37 active compounds^2^ are registered for *B. cinerea* control. They include several copper-based fungicides, folpet, cyprodinil, but also *Bacillus amyloliquefaciens* sp. *plantarum* and *Bacillus subtilis* for organic production. Tomato is one of the most important vegetable crops worldwide and is situated among the top 10 in terms of yield (fresh weight)^3^. The aim of this study was to identify tomato leaf infection by *B. cinerea* using (low cost) multispectral imaging allowing an earlier infection recognition compared to visual detection. Specifically, we investigated (1) what time after *B. cinerea* infection allows a discrimination of healthy and diseased leaf tissue?; and (2) which of the multispectral bands are the most informative for disease detection?

## Materials and Methods

Time lapse experiments were conducted and repeated three times. In brief, tomato leaflets were pathogen- or mock-inoculated for 5 h as described elsewhere (Rezzonico et al., 2017). The inoculum drops were removed with a paper towel and the leaflets imaged with a multispectral imager in regular intervals until 30 hpi. Data extracted from the imagery was used to separate healthy and infected tissue.

### Plant Material

Tomato plants (*S. lycopersicum*, Heinz 1706 cultivar) were grown in standard soil (Floradur^®^ Block Bio, Floragard, Oldenburg, Germany) in a semi-regulated greenhouse with open windows. The temperature was set to 20–26°C with maxima during sunny summer days of up to 40°C. On cloudy days, artificial light was used to achieve minimal constant lighting of 80 kW per square meter for 16 h per day. Once a week, cuttings were produced from tomato mother plants, that were treated weekly with sulfur (Stulln WG, Andermatt Biocontrol, Grossdietwil, Switzerland). The cuttings were then placed in approximately 100% relative humidity for 1 week to develop roots. Afterward, they were acclimatized to the same greenhouse conditions mentioned above. Young and fully unfolded leaflets were harvested from two-week old cuttings for inoculation trials. For inoculation experiment five leaflets were placed on agar petri dishes (1% w/v; water only). The leaflets of each six petri dishes were drop-inoculated with pathogen or mock inoculum suspension as described below.

### Inoculum Preparation, Inoculation and Sampling

A *B. cinerea* strain T4 was grown on 15 g/l malt agar (Fluka, Sigma-Aldrich, Buchs, Switzerland) plates for 3–8 weeks. Spores were harvested with 20 ml half-strength grape juice (Farmer, Landi, Dotzigen, Switzerland) and diluted to 1.3 × 10^6^ spores per ml. The spore suspension was used directly for inoculation with one to three 10-ul-drops placed on the abaxial surface of each leaflet. The inoculated leaves were stored at 18°C, 80% rel. humidity, and 16 h light but without light for the first 24 hpi. Mock inoculations were performed under the same conditions using half-strength grape juice for inoculation. The success of infections was recorded at 30 hpi.

### Image Acquisition

The time lapse experiments were conducted in a growth chamber (Fitotron SGC 120, Weiss Technik, Altendorf, Switzerland) that was equipped with additional lighting (two 400 W halogen incandescent lights, 0.3 × 0.3 m diffusion paper, Supplementary Figure S1) to ensure uniform illumination conditions during image acquisitions. Images were taken with a MicaSense^®^ RedEdge3^TM^ (Seattle, WA, United States) multispectral camera collecting 10–40 nm-wide bands in blue, green, red, near-infrared, and rededge (Table 1). Each band of multi-lense MicaSense^®^ RedEdge3^TM^ camera records 1280 × 960 pixels (1.2 Mpixels). In the experimental setup described, each pixel covers approximately 0.2 mm^2^. For each time lapse experiment twelve petri dishes with pathogen- and mock-inoculated leaflets were positioned below the camera with a distance of 66.6 cm (Supplementary Figure S2). Six petri dishes were positioned with an angle of 90° and another six with approximately 64° toward the sensor. Camera and lights were triggered using a Python v2.7 script to collect imagery. The automated procedure switched lights of the growth chamber off and halogen lights on at each sampling time. Time lapse image acquisition started 05:00, 05:15, and 05:45 hpi. Images were taken every 80 min in 17, 20, and 19 frames (approximately until 29 hpi), each with 16, 16, and 15 image repetitions per frame leading to a total of 272, 320, and 285 multispectral images (Table 2). Images were recorded in 12-bit RAW format and converted to 16-bit TIFF format prior to processing. The sensor signal values increase almost linearly to input radiance. To reduce the amount of noise in the images we used low gain values and shutter speeds around 5–20 ms. After time lapse experiment at 30 hpi the status of infection was recorded with a hand held RGB camera (Sony DSC RX100IV, 20 Mpixels). These images were used to locate the position of infection (regions of interest, ROI) drops by manual identification of the necrotic lesions.

**Table 1:** Spectral bands with center wavelength and bandwidth of the MicaSense RedEdge multispectral camera^1^.

Band		Center wavelength	Band width
number	Band color	(nm)	(nm)
1	Blue (B)	475	20
2	Green (G)	560	20
3	Red (R)	668	10
4	Near infrared (NIR)	840	40
5	Rededge (RE)	717	10

*^*1*^https://support.micasense.com/hc/en-us/article_attachments/204648307/RedEdge_User_Manual_06.pdf accessed 18.10.2018.*

**Table 2:** Key data of the time lapse experiments (hpi: hours post inoculation).

	Time lapse 1	Time lapse 2	Time lapse 3
	26.07.2017	01.09.2017	04.09.2017
Start (*hh:mm*, hpi)	05:00	05:15	05:45
Image acquisitions duration (*hh:mm*)	22:40	26:40	25:20
No. frames	17	20	19
No. images/frame	16	16	15
Σ images/band (of all 5 bands)	272 (1369)	320 (1600)	285 (1425)
Total experiment duration (*hh:mm* pi)	27:40	31:55	31:05

### Image Processing Procedure

**FIGURE 1:**
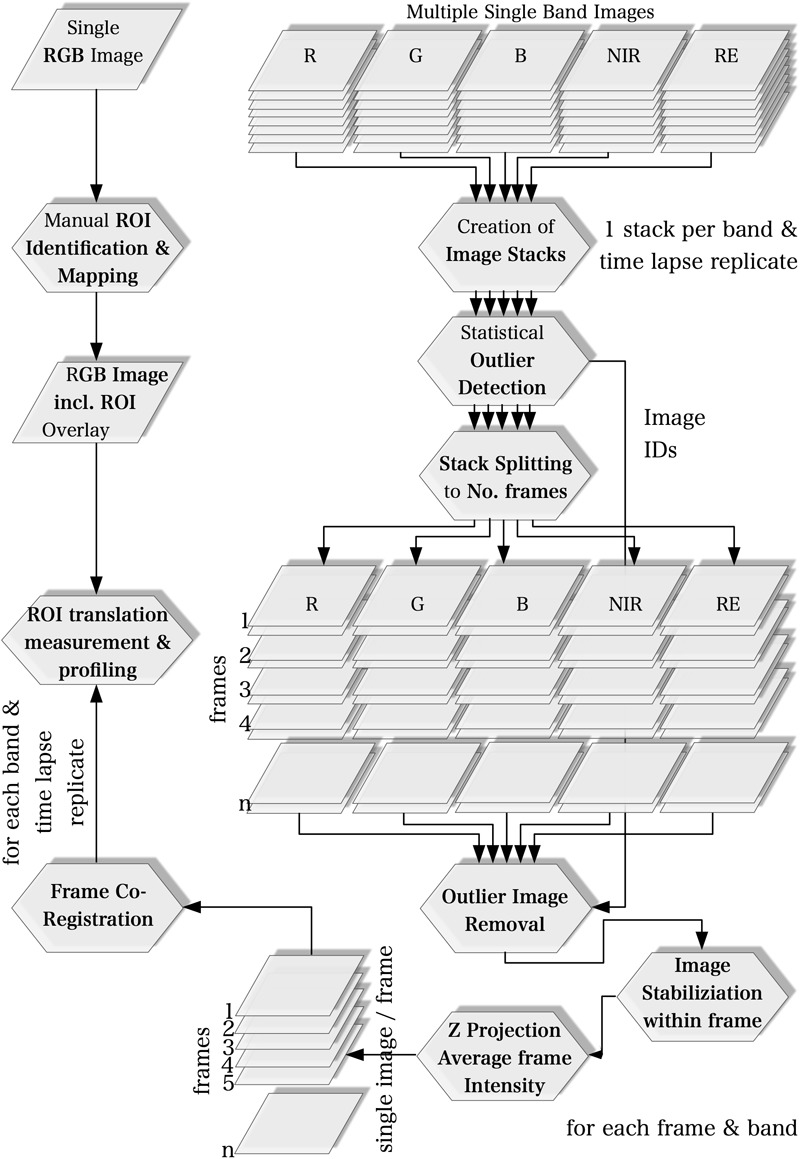
Workflow of image acquisition.

We used the ImageJ software (v. 1.51) bundled in the FIJI distribution (Schindelin et al., 2012) for image processing and time lapse analysis. The multi-lens and multi-sensor geometry of the camera model, i.e., differences in mounting position and viewing angles among lenses, causes significant band misregistration effects. Thus, the creation of multispectral images requires band co-registration. Band to band registration is, however, a computationally intensive process. Our workflow, therefore, is based on the idea to process every spectral band individually. Instead of registering the image bands to each other to compose multispectral images, we only compute the matrices to translate the ROI locations from a common reference to their individual location on each image band. The workflow basically consists of five steps which are described in detail in the following sections and summarized in Figure 1. Only the first step was applied to a single multispectral image with all five bands while all other processing steps were executed only on the individual spectral bands.

#### ROI Identification on a Common Reference (all Bands)

Regions of interest positions on pathogen infected leaflets were identified manually on RGB images taken 30 hpi. On the same images one ROI position on each mock-inoculated leaflet was defined manually from the positions of mock-inoculum drops. ROIs were drawn on the RGB images using the *Oval Selection Tool* and labeled within the *ROI Manager.* A 5 px circular shape was used to represent the ROIs covering 12 complete image pixels.

#### Stack Creation and Statistical Outlier Image Detection (Band-Wise)

Image stacks were created for each spectral band and the mean intensity of every image and of the entire stack was calculated to identify images whose intensity differs significantly from the intensity range of the other images. An image is considered a statistical significant outlier if the difference of the mean intensity of the image to the mean intensity of the stack is greater than 2 times the standard deviation of the stack intensity. In time lapse replicate one (20170726) no outlier images were found. In time lapse replicate two (20170901) and three (20170904) 24 (7.5%) and 17 (6%) outlier images were identified, respectively.

#### Image Stabilization, Outlier Image Removal and Calculation of Mean Image Intensities on the Frame Scale (Band-Wise)

Image stacks were split to the number of frames and the number of images in each stack equals the images repetition rate. Outlier images identified in step 2 were deleted and image alignment of each stack was optimized with reference to the first image of the frame using the *Image Stabilizer* plugin^4^. Then, all images of each frame were aggregated to a single image representing the average intensity of that frame.

#### Frame Co-registration on the Scale of the Time Lapse Experiment (Band-Wise)

The average intensity images of each frame were stacked again and co-registered to the first image of the frame using a rigid 2D transformation model in the *Descriptor-based registration* plugin (Preibisch et al., 2010). While step 3 optimized image alignment at the frame scale, step 4 aimed at co-registering the frames to each other and as such at optimizing the frame displacement to the scale of the entire time lapse. After completion of step 4 the number of images in the stacks equals the number of frames of the time lapse experiments with frame displacements averaged over all five bands of 0.31 ± 0.03 px, 0.34 ± 0.02 px, and 0.31 ± 0.03 px for time lapse replicate one, two and three, respectively.

#### ROIs Translation and Multiple ROI Measurement and Profiling (Band-Wise)

To translate the ROIs defined in step 1, we co-registered the RGB images including the ROI overlays to the last frame of every band. For this purpose, the robust and elastic 2D image registration method presented by Wang et al. (2014) was used. The co-registered RGB images then served as templates on which the ROIs were traced and re-drawn using the *Oval Selection Tool*.

#### Background ROI

**FIGURE 2:**
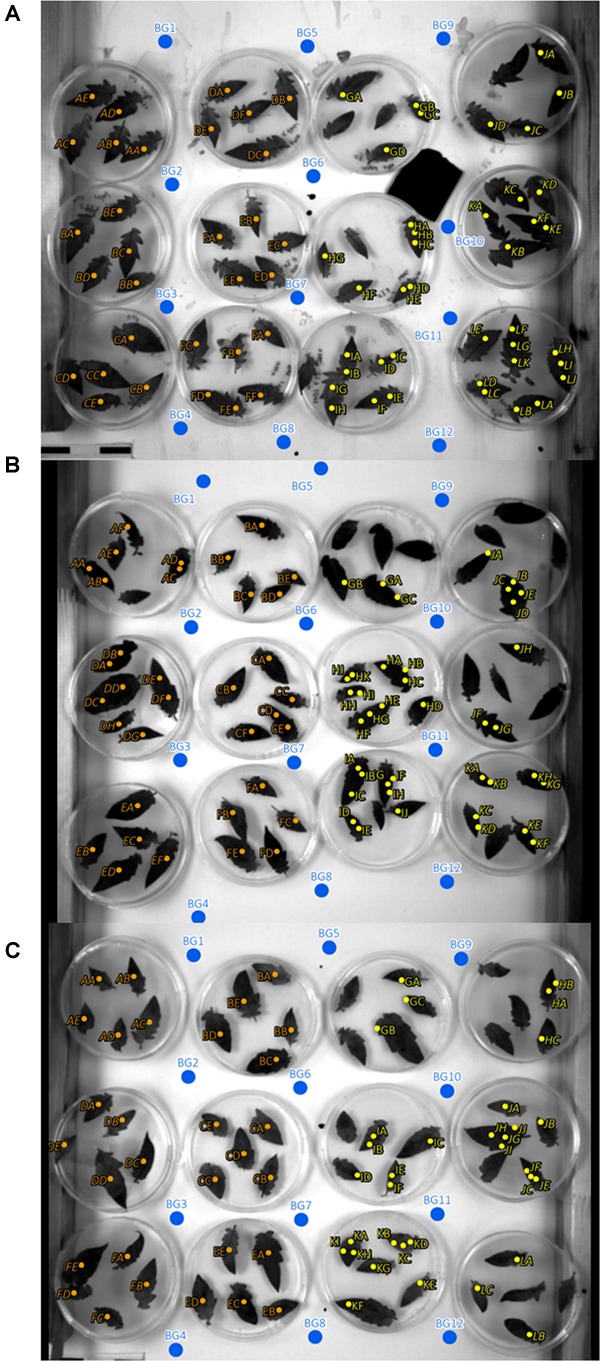
Regions of interest (ROIs) and background ROI locations of experiments 1, 2, and 3 **(A–C)**. Orange, mock inoculated; yellow, pathogen infected; blue, background ROIs; italic letters, inclined leaves with an angle to sensor of approximately 64°; non-italic letters, 90° angle to sensor.

In addition to the ROI positions on mock- and pathogen-inoculated leaflets, twelve ROIs covering background only where defined (Figure 2). The intensity of the background ROIs was used to exclude any bias caused by illumination or camera artifacts in relation to leaflet position within the experimental setup.

### Classification of Infected and Healthy Tissue

An unsupervised classification scheme coupled with a masking approach was used to identify infected and healthy sections of the leaflets. At first, circular selections were drawn around petri dishes and attributed with dish IDs so that calculations could be performed for each petri dish individually. We created binary image masks of the leaflets to exclude any background information and to make use of image pixels that represent either healthy or diseased tissue. The binary leaf masks were computed from the red band images using the Minimum Cross Entropy thresholding method as developed by Li and Tam (1998) and implemented in the *Auto Threshold plugin* of the ImageJ software. Binary leaflet masks were then assigned to the NIR and RE bands in the same way as the ROIs [c.f. see section “ROIs Translation and Multiple ROI Measurement and Profiling (Band-Wise)”]. The binary masks basically black and white pictures having an inverting Lookup table LUT with values of 0 and 1. Multiplying the NIR and RE stacks with these masks results in bit-masked version of the stacks. Next, we applied an iterative self-organizing (ISO) classifier to separate the masks of the image into two classes (unsupervised). The class with the lower values was supposed to correlate with the infected parts of the leaflets and *vice versa*.

### Statistical Analysis

We developed linear regression models to predict the temporal change in band intensity (independent variable) captured with multispectral imager. The intensity was normalized (i.e., division by maximum value) separately for each band and trial. Observation time used as dependent variable was scaled prior analyses (i.e., subtracted by mean and divided by standard deviation).

The data sets have two characteristic properties that are of great importance for the statistical analysis. First, the measured band intensities represent time dependent observations. To account for temporal dependence, we used a parametric bootstrap approach (Efron and Tibshirani, 1994) to derive 95% confidence intervals (from 2.5 to 97.5% quantiles of the simulated distributions) for both the regression parameters and the measures of model fit: R^2^ (coefficient of determination) and RMSE (root mean squared error). We used a fixed-x resampling approach, meaning that we sampled with replacement from the original residuals in each iteration. The number of iterations was set to 1000.

Second, the semi-random distribution of petri dishes which may violate the assumption of independent residuals. To show that the experimental design (Figure 1) has no effect on parameter estimates, we performed additional linear regressions using (a) a 10-fold cross validation, and (b) parametric stratified bootstrapping. For the stratified bootstrapping approach, we used band intensities of background points as dependent variables (Figure 2). Our hypothesis was that if sampling time was unrelated to those background (or control) intensities that we can exclude an influence of our experimental design on the regression outcome. Intensity of control samples were drawn with replacement using the entire original sample or within one of following two strata within the original data set: (1) horizontally, and (2) vertically distributed petri dishes, respectively.

## Results

**FIGURE 3:**
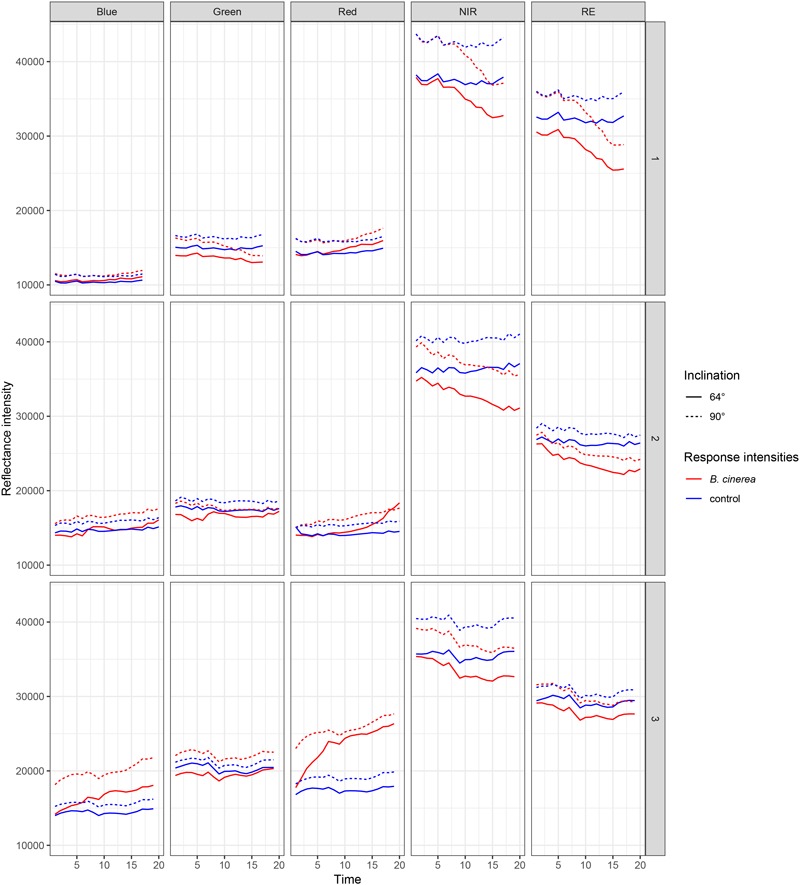
Reflectance intensities of bands blue, green, red, NIR (near-infrared), and RE (rededge) collected from ROIs of *B. cinerea*-infected (BC) and mock-inoculated (control, BCm) tomato leaves in experiments 1, 2, and 3.

In three replicated experiments, first, leaflets were pathogen- and mock-inoculated, and subsequently, imaged continuously every 80 min with a five-lens multispectral camera. Raw reflectance intensity (digital numbers) varied for red (R), blue (B), and green (G) between approximately 15000 and 28000, near-infrared (NIR) reached intensities of 42000 and rededge (RE) of 35000 (Figure 3). Over time, red is increasingly reflected from pathogen infected leaf tissue whereas NIR and RE reflectance is decreasing. In band R, NIR, and RE the differences in reflectance intensities between mock- and pathogen-inoculated samples were increasing over time, and hence, these three bands were more informative than G and B (Table 3 and Figure 3).

**Table 3:** Summary statistics of *R*^2^ and root mean squared error (RMSE) of linear regressions to predict multispectral band intensity (bands one to five) of either *B. cinerea* strain T4 (pathogen, Type1) or mock (control, Type 0) inoculations of tomato leaves.

		*R*^2^	*R*^2^	*R*^2^	RMSE	RMSE	RMSE
Band	Inoculation	mean	2.5%	97.5%	mean	2.5%	97.5%
Blue	Pathogen	0.46	0.37	0.55	0.066	0.0585	0.0739
Green	Pathogen	0.45	0.36	0.54	0.0499	0.0441	0.0558
Red	Pathogen	0.64	0.56	0.71	0.0462	0.0403	0.0515
NIR	Pathogen	0.87	0.84	0.89	0.0252	0.0223	0.0281
RE	Pathogen	0.45	0.36	0.54	0.0615	0.0545	0.0678
Blue	Mock	0.06	0.02	0.13	0.132	0.118	0.147
Green	Mock	0.46	0.37	0.55	0.0330	0.0293	0.0370
Red	Mock	0.09	0.03	0.16	0.120	0.107	0.131
NIR	Mock	0.88	0.86	0.91	0.0196	0.0175	0.0216
RE	Mock	0.65	0.58	0.71	0.0226	0.0200	0.0250

**FIGURE 4:**
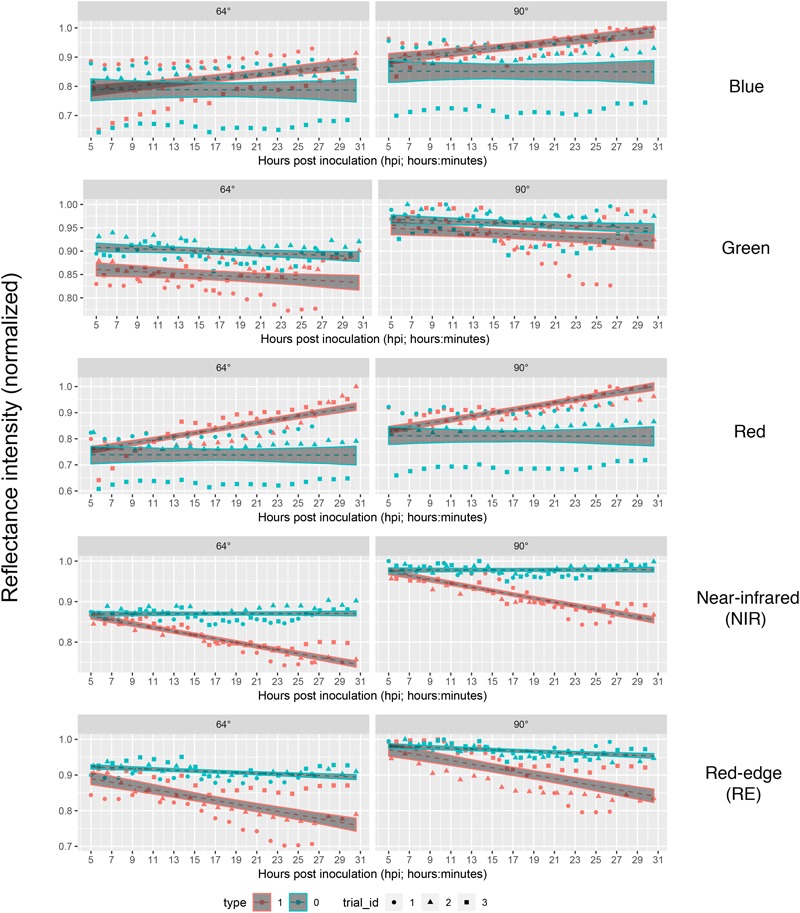
Linear predictions of multispectral band intensity (1–5) for *B. cinerea* strain T4 (pathogen, Type1) and mock (control, Type 0) inoculations of tomato leaves depending on the hours post inoculation and different petri dish inclinations (64° vs. 90°). Type 1, *B. cinerea*-infected; type 2, mock-inoculated; trial_id; trial_id, replicate of experiment.

Each series of images was analyzed separately per repetition and band by means of linear regression (Figure 4) without considering the different leaf to lens angles. With the regression coefficients for the dependent variable “time” being insignificant (*p* > 0.05), predictions of reflectance intensities for band blue and red were constant for mock-inoculated leaflets (Tables 3, 4). In contrast, we found a significant increase for pathogen-infected leaflets for the blue (mean estimate = 0.0260; *p* < 0.001) and red (mean estimate = 0.0473; *p* < 0.001) band. The reflectance intensity of the green band was decreasing over the whole experiment in pathogen- and mock-inoculated leaflets. In contrast to the blue and red band, no significant relation was found between NIR and RE reflectances of pathogen-infected leaflets. The intensity of NIR was constant (i.e., the mean estimate had a *p*-value > 0.05) in healthy leaflets. In the pathogen-infected leaflets, the intensity of the red band was slightly but significantly decreasing. Confidence interval and RMSE of NIR reflectance intensities of both healthy and diseased leaflets were smallest and had an *R*^2^ = 0.85 indicating the lowest variability between experimental bootstraps and highest explanatory power (Table 3).

**Table 4:** Summary statistics of *R*^2^ and root mean squared error (RMSE) of linear regressions to predict multispectral band intensity (bands one to five) of either *B. cinerea* strain T4 (pathogen, Type1) or mock (control, Type 0) inoculations of tomato leaves.

		R^2^	R^2^	R^2^	RMSE	RMSE	RMSE
Band	Infection	mean	2.5%	97.5%	mean	2.5%	97.5%
Blue	Pathogen	0.50	0.40	0.58	0.0425	0.0374	0.0477
Green	Pathogen	0.15	0.08	0.24	0.0465	0.0410	0.0519
Red	Pathogen	0.31	0.22	0.41	0.0694	0.0617	0.0767
NIR	Pathogen	0.85	0.82	0.88	0.0269	0.0235	0.0304
RE	Pathogen	0.54	0.45	0.62	0.0459	0.0404	0.0513
Blue	Mock	0.19	0.11	0.29	0.0696	0.0619	0.0772
Green	Mock	0.59	0.51	0.67	0.0255	0.0228	0.0285
Red	Mock	0.17	0.09	0.26	0.0823	0.0738	0.0910
NIR	Mock	0.88	0.86	0.91	0.0196	0.0175	0.0216
RE	Mock	0.64	0.56	0.70	0.0234	0.0208	0.0259

*In contrast to Table 3, this table confers to data with an adjusted start value considering the leaf tissue to be healthy at 5 hpi and hence equalizing the first measurement value of mock- and pathogen infected ROIs.*

The intensity of leaflets positioned at a 90° angle toward the sensor was generally higher than with a smaller angle of 64° (Figure 3). In the NIR band the difference was slightly larger. Differences in reflectance intensity between mock- and pathogen-inoculated samples remained almost the same independently of leaf-camera angle.

Taking the visual diagnosis of the overlap of the pathogen and mock infected confidence intervals of the linear predictions as a measure which allows to select a time at which mock-inoculated tissue could be differentiated from pathogen-infected one, we found that time point 4 (corresponding to 9 to 9:45 hpi) should be the earliest possible time instant in bands red, NIR and RE taking both angles into account (Figure 4).

**FIGURE 5:**
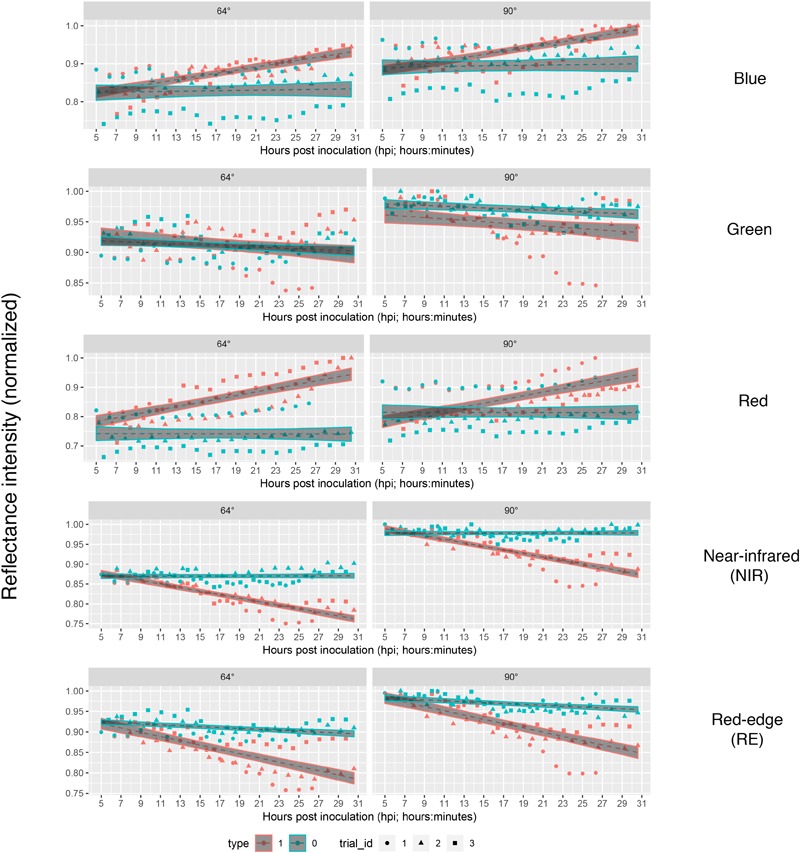
Linear predictions of multispectral band intensity as in figure three with an adjustment of first sampling time. The value of mock- and pathogen-inoculated samples of first sampling time was set equal to imitate the leaf tissue to be healthy at the first sampling time at approximately 5 hpi. Type 1, *B. cinerea*-infected; type 2, mock-inoculated; trial_id, replicate of experiment.

If we assume the infected samples at the first measurement time to be healthy (approximately at 5 hpi), the intensities of reflectance of mock- and pathogen infected tissue are shifted toward each other (Figure 5). This normalization allowed an enhanced discrimination of mock- and pathogen-inoculated ROIs at measuring time 5 (corresponds to 10:20–11:05 hpi). In regard of mean *R*^2^ (pathogen: NIR 0.85 vs. RE 0.54, mock: NIR 0.88 vs. RE 0.62) and mean RMSE (pathogen: NIR 0.0269 vs. RE 0.0459, mock: NIR 0.0196 vs. RE 0.0234) NIR outperforms RE (Table 4). The coefficients of linear regression of NIR band of pathogen infected ROIs were significantly influenced by time in contrast to the corresponding mock-inoculated ROI-derived values (Tables 5, 6). Time-caused effects were not found in background ROIs and reflectance intensities in the background ROIs differed not significantly (Supplementary Figure S2). Therefore, any bias influencing the resulting data caused by experimental setup or sensor artifacts could be excluded.

**Table 5:** Summary statistics of parameter estimates of linear regressions to predict multispectral band intensity (bands one to five) of either *B. cinerea* strain T4 (pathogen, Type1) or mock (control, Type 0) inoculations of tomato leaves.

Band	Infection	Parameter	Estimate	Std. error	*p*-value
Blue	Pathogen	Intercept	0.829	8.94E-03	<0.001
Blue	Pathogen	Time	0.0260	6.32E-03	<0.001
Blue	Pathogen	Inclination (90)	0.109	0.0126	<0.001
Green	Pathogen	Intercept	0.848	6.77E-03	<0.001
Green	Pathogen	Time	-7.91E-03	4.79E-03	0.170
Green	Pathogen	Inclination (90)	0.089	9.57E-03	<0.001
Red	Pathogen	Intercept	0.836	6.25E-03	<0.001
Red	Pathogen	Time	0.0473	4.42E-03	<0.001
Red	Pathogen	Inclination (90)	0.0772	8.85E-03	<0.001
NIR	Pathogen	Intercept	0.808	3.41E-03	<0.001
NIR	Pathogen	Time	-0.0337	2.41E-03	<0.001
NIR	Pathogen	Inclination (90)	0.109	4.82E-03	<0.001
RE	Pathogen	Intercept	0.828	8.34E-03	<0.001
RE	Pathogen	Time	-0.0371	5.90E-03	<0.001
RE	Pathogen	Inclination (90)	0.0814	0.0118	<0.001
Blue	Mock	Intercept	0.788	0.0179	<0.001
Blue	Mock	Time	-6.86E-04	0.0127	0.619
Blue	Mock	Inclination (90)	0.0626	0.0253	<0.05
Green	Mock	Intercept	0.899	4.48E-03	<0.001
Green	Mock	Time	-5.64E-03	3.17E-03	0.145
Green	Mock	Inclination (90)	0.0600	6.33E-03	<0.001
Red	Mock	Intercept	0.738	0.0164	<0.001
Red	Mock	Time	-4.99E-04	0.0115	0.608
Red	Mock	Inclination (90)	0.0725	0.0230	<0.01
NIR	Mock	Intercept	0.870	2.65E-03	<0.001
NIR	Mock	Time	3.64E-04	1.87E-03	0.590
NIR	Mock	Inclination (90)	0.108	3.75E-03	<0.001
RE	Mock	Intercept	0.909	3.06E-03	<0.001
RE	Mock	Time	-8.05E-03	2.17E-03	<0.01
RE	Mock	Inclination (90)	0.0588	4.33E-03	<0.001

*Estimates, standard errors and *p*-values represent mean values of 1000 bootstrap iterations.*

**Table 6:** Summary statistics of parameter estimates of linear regressions to predict multispectral band intensity (bands one to five) of either *B. cinerea* strain T4 (pathogen, Type1) or mock (control, Type 0) inoculations of tomato leaves.

Band	Infection	Parameter	Estimate	Std. error	*p*-value
Blue	Pathogen	Intercept	0.875	5.76E-03	<0001
Blue	Pathogen	Time	0.0305	4.07E-03	<0001
Blue	Pathogen	Inclination (90)	0.0579	8.14E-03	<0001
Green	Pathogen	Intercept	0.913	6.30E-03	<0001
Green	Pathogen	Time	-8.27E-03	4.45E-03	0.126
Green	Pathogen	Inclination (90)	0.0346	8.90E-03	<001
Red	Pathogen	Intercept	0.860	9.40E-03	<0001
Red	Pathogen	Time	0.0458	6.65E-03	<0001
Red	Pathogen	Inclination (90)	-1.05E-03	0.0133	0.613
NIR	Pathogen	Intercept	0.822	3.64E-03	<0001
NIR	Pathogen	Time	-0.0325	2.58E-03	<0001
NIR	Pathogen	Inclination (90)	0.112	5.15E-03	<0001
RE	Pathogen	Intercept	0.857	6.22E-03	<0001
RE	Pathogen	Time	-0.0380	4.40E-03	<0001
RE	Pathogen	Inclination (90)	0.0629	8.79E-03	<0001
Blue	Mock	Intercept	0.829	9.43E-03	<0.001
Blue	Mock	Time	2.52E-03	6.67E-03	0.572
Blue	Mock	Inclination (90)	0.0662	0.0133	<0.001
Green	Mock	Intercept	0.9111	3.46E-03	<0.001
Green	Mock	Time	-4.74E-03	2.45E-03	0.116
Green	Mock	Inclination (90)	0.0603	4.89E-03	<0.001
Red	Mock	Intercept	0.741	0.0112	<0.001
Red	Mock	Time	-3.46E-04	7.89E-03	0.604
Red	Mock	Inclination (90)	0.0729	0.0158	<0.001
NIR	Mock	Intercept	0.870	2.65E-03	<0.001
NIR	Mock	Time	3.64E-04	1.87E-03	0.590
NIR	Mock	Inclination (90)	0.108	3.75E-03	<0.001
RE	Mock	Intercept	0.910	3.17E-03	<0.001
RE	Mock	Time	-7.96E-03	2.24E-03	<0.01
RE	Mock	Inclination (90)	0.0589	4.49E-03	<0.001

*In contrast to Table 5, this table confers to data with an adjusted start value considering the leaf tissue to be healthy at 5 hpi and hence equalizing the first measurement value of mock- and pathogen infected ROIs. Estimates, standard errors and *p*-values represent mean values of 1000 bootstrap iterations.*

**FIGURE 6:**
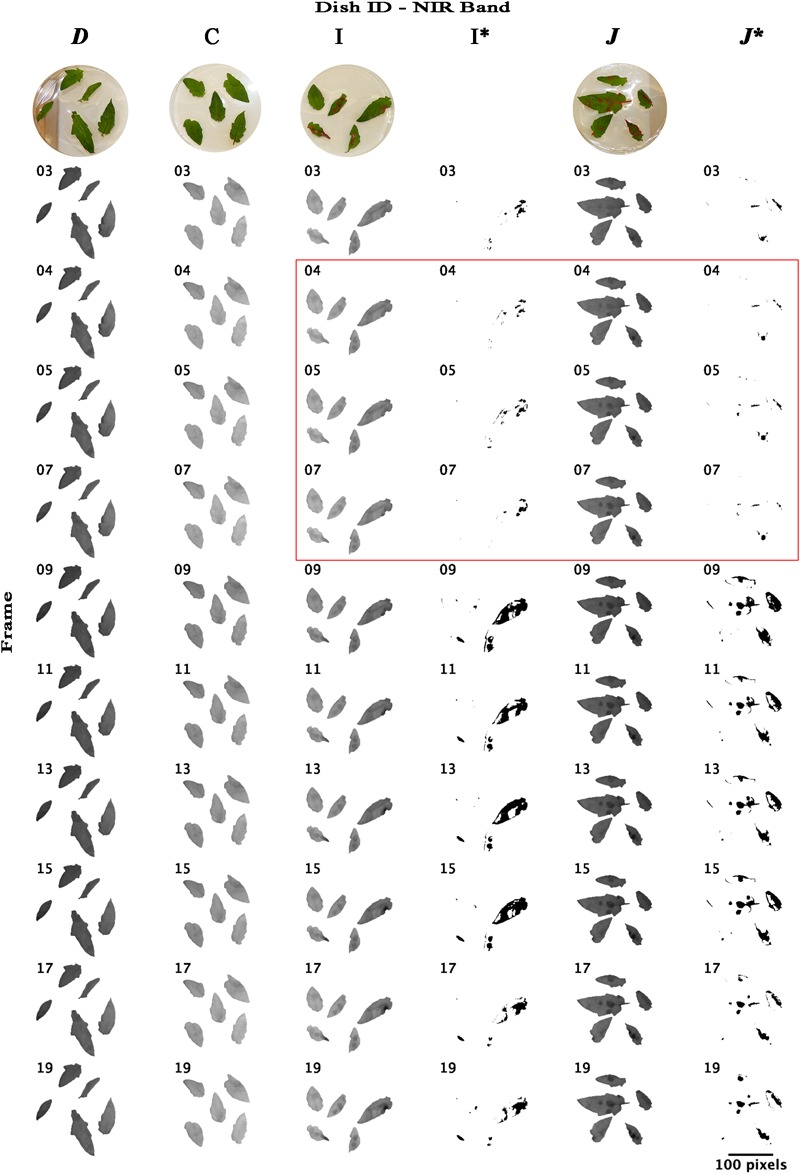
Time lapse stack montages of NIR (near-infrared) reflectance intensities of mock-inoculated and *B.* cinerea-infected tomato leaves of trial 3. The sequence from left to right corresponds to both, infection type and petri dish inclination and includes the dishes *D* (mock inoculated, 64° inclination), C (mock inoculated, 90° angle to sensor), I (pathogen infected, 90°) and *J* (pathogen infected, 64°). Montages I^∗^ and *J^∗^* represent binary masks of approximated locations of pathogen infection (black cells) derived from unsupervised ISO data classification. First line, RGB images taken at approximately 30 hpi; red labels indicate the infected and damaged leaf area. Red square highlights the petri dishes photographed at measurement time 4, 5, and 7. Red square indicates the leaflets on petri dishes. Measurements were conducted in 80 min intervals. Measurement times 3, 4, 5, … correlated to 8:25, 9:45, 11:05,…, hpi. Size bar corresponds to 100 pixel which correlates to approximately 7 cm.

**FIGURE 7:**
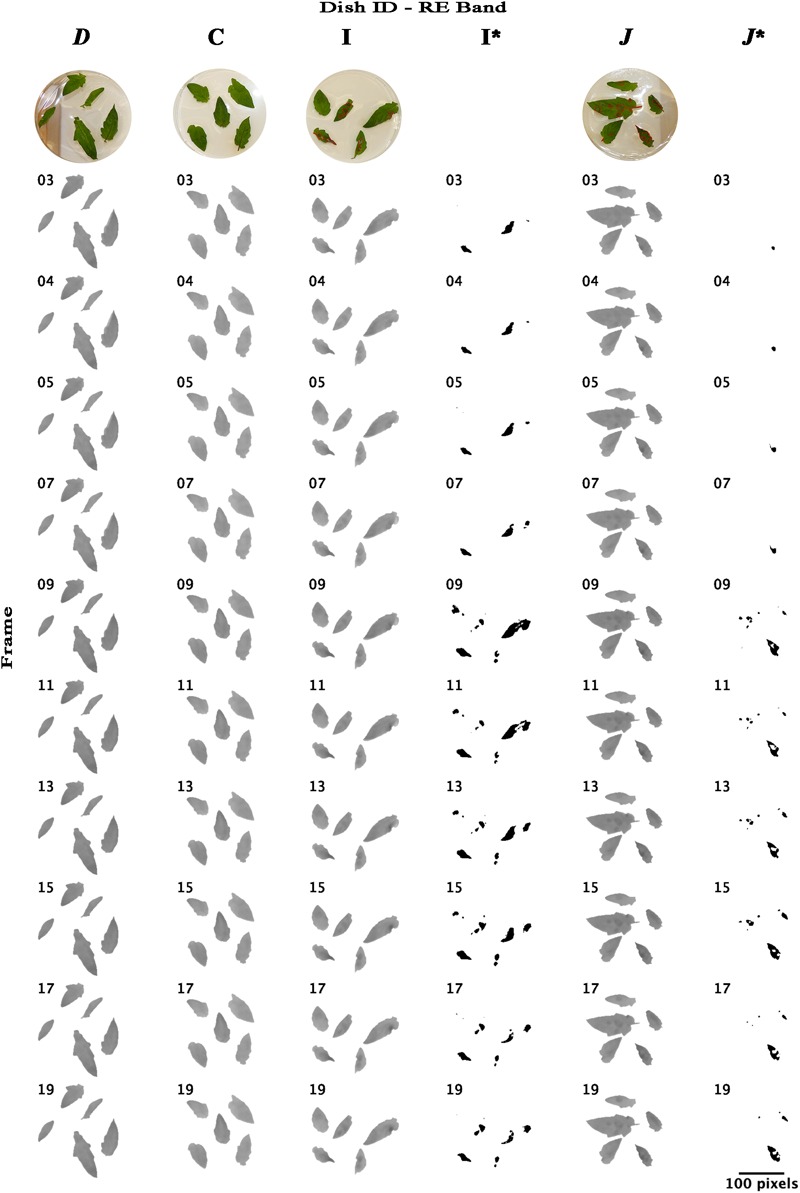
Time lapse stack montages of RE (rededge) reflectance intensities of petri dishes *D*, C, I, and *J*. Data sequence and figure structure is the same as in Figure 6. Size bar corresponds to 100 pixel which correlates to approximately 7 cm.

Re-analyzing the images of bands NIR and RE with an unsupervised classification using imageJ software that forms patches of pixels containing distinct classes (class “mock-inoculated” with higher reflectance intensities than class “pathogen-infected”), we were able to classify potentially diseased and healthy leaf areas and approximately locate pathogen infection spots (Figure 6, 7). In NIR imagery of measurement times 3, 4, and 5 (correlating to 8:25, 9:45, and 11:05 hpi) infection locations became slightly visible (Figure 6, petri dish I and *J*). Correlating classifications (I’ and *J’*) became visible from 9:45–13:45 hpi and artifacts on the leaf borders from measurement time 9 (16:25 hpi) onward. The classification of RE images located diseased spots at 16:25 hpi (Figure 7). Comparing potential symptoms manually in NIR and RE at 9:45 hpi images to necroses visible on RGB imagery taken at approximately 30 hpi with the classifications of 16:25 hpi, an overlap of diseased and healthy regions was detected qualitatively. However, quantitative results from NIR and RE imagery classification were blurred due to classification artifacts at the ribs and the borders of the leaves. Including the potential artifacts, the percentage of necrotic leaf surface detected with NIR and RE reached up to 43% and 35% in 90° leaf angles, and 42% and 11% in 63° leaf angels, respectively (Supplementary Table S1). However, these maxima appear at 16:25 hpi and correlated with the visible artifacts.

## Discussion

This paper aimed at demonstrating the application of low cost multispectral sensors as a useable tool to identify leaf pathogen infection at a pre-visual stage instead of using the naked eye only. We exemplified the application of this early screening approach using the necrotrophic fungus *B. cinerea* under laboratory conditions. The used multispectral imager was a five-band multi-lens sensor. Our findings suggest that narrow band sensors in the NIR range are suited to detect fungal disease attack at a pre-visual stage. Reflectance of leaves in both tested angles (90° and 64°) could be sufficiently analyzed with only one band, indicating that a one-band sensor would be sufficient to detect the disease. For background masking and subtraction, however, additional bands such as red and green might be useful. If several band of a multi-lens sensors are needed an accurate band co-registration (i.e., the translation and rotation of the images, that each corresponding pixel refers to the same location of the imaged object) increases computing steps and time at least by the factor of band used. Therefore, single-lens multispectral camera models would be useful alternatives. In contrast to LED-based multispectral imagers (Grieve et al., 2015), cameras with filters allowing only narrow bands of light to pass, might be useful under real-world conditions, because they do not rely on close proximity, darkness and additional LED light source.

We measured increasing reflectance intensity in the red and blue band, a relatively constant reflectance in the green band, and a decreasing reflectance in the NIR and RE band (Figure 3). This is consistent with findings by Mahlein et al. (2010) who found similar infection patterns studying sugar beet leaves infected with fungal diseases such as *C. beticola*- and *U. betae*. In contrast, Zhao et al. (2014) found an increased reflectance intensity in the NIR range for *Puccinia striiformis* infections which cause stripe rust in wheat. The spectral signatures in barley leaves were found to be increased in the measured range from 400 to 2400 nm when infected with net blotch, brown rust or powdery mildew (Thomas et al., 2018). Additionally, the reflectance intensities were found to be growth stage dependent (Zhao et al., 2014).

Under laboratory conditions using *B. cinerea* strain T4 as an example fungal pathogen, we were able to identify differences in linear regression models of healthy and diseased tissue as early as 9 hpi with the NIR band. The NIR band filter is a 40 nm wide band with a center wave length of 840 nm. The NIR band had the smallest confidence interval while separating mock-and pathogen-infected ROIs the earliest. This finding confirms qualitatively the results of Xie et al. (2017) who plotted hyperspectral data of *B. cinerea*-infected and healthy tissue. Their results indicate 746 nm to be the most informative wavelength. The RE band collects reflectance irradiance around 717 nm. RE is the region in which the reflectance intensity increases the most (Xie et al., 2017), what may explain the bigger variability than in the NIR range.

We analyzed the reflectance of pathogen and mock-inoculated tissue by comparing defined ROIs on healthy and disease-attacked leaf tissue. Under real-world-conditions varying angles of sun and leaf position to camera cause varying reflectance intensities (Pinter et al., 1985; Maes et al., 2014; Oberti et al., 2014). Therefore, transferring this artificial setup to field conditions, the differences of reflectance from one same leaf should be considered only. We investigated two angles (90° and 64°) the leaves were positioned toward the camera sensor. Using hyperspectral imaging of *Erysiphe necator* infected vine leaf tissue under laboratory conditions, Oberti et al. (2014) indicated that angles smaller than 90° lead to better differentiation of diseased and healthy tissue. The importance of leaf angle for disease detection by hyperspectral imaging was confirmed by our results in wavebands RE and NIR. For both bands the smaller angle lead to smaller and earlier separated confidence intervals of reflectance intensity. To this end, more work should clarify the reasons for it.

Natural infection does usually not occur in spots comparable to the artificial drop infection we used in our experiment but from smaller infection sites and maybe from single infection units. On the one hand, this may lead to a delay of detection, since infected tissue patches are smaller as the experimental ROIs and resolution of the sensor is limited. On the other hand, fungal infections may lead to specific shapes that should be investigated in future studies. Moreover, the differences in reflectance intensity may be also induced by other biotic and abiotic stresses. For instance, water deficit in tomato leads to a lower reflectance intensity than well-watered tomato plants (Susiè et al., 2018). Water deficit cause differently shaped patches or even impact whole leaves only compared to fungal infections. Different shapes in combination with intensity may be key to separate biotic and abiotic stress. The potential artifacts we found by classifying diseased from healthy tissue (Figure 6, 7), may be caused by abiotic stress induced by, e.g., the used experimental setup with detached leaves. Abiotic and additional biotic stresses should be addressed in future experiments to validate specificity of reflectance data.

Due to technical constrains, we used a semi-randomized experimental setup. However, we excluded any bias caused by the setup or sensor artifacts with a cross validation of all ROIs and a stratified bootstrapping of the background ROIs. The factor “time” was found to be not significant in the stratified bootstrapping. Therefore, we can exclude any bias in the linear regressions caused by the experimental design. The observed differences in reflectance intensities at the first measurement point (5 hpi) were most prominent in red and blue bands of the third experiment. This could have been potentially caused by camera or light setting shifts. However, this was a systematic error which was addressed by normalization.

Unsupervised classification using the imageJ software was of limited success compared to linear regression results. Diseased tissue could be identified in the NIR images from 9:45–13:45 which correlates to the results of linear regression. Artifacts like leaf borders and veins became visible from 16:25 hpi onward. This may be due to tissue aging and beginning senescence because we were using a detached leaf assay. Considering potential specific spatial shapes of biotic and abiotic stresses (i.e., for example, circular expanding reflectance intensity changes around infection location vs. changes along the leaf-veins) and taking into account the low resolution snap-shot multispectral imagers available, logic pattern-based Logical Vision machine learning approaches (Muggleton et al., 2018) may be useful tools for collecting information from low resolution multispectral images. In addition, demonstrating reflectance changes within the first 24 h of fungal infections may correlate to reported drastic changes on gene expression level. In *Arabidopsis thaliana* challenged with *B. cinerea* several hundred genes were differentially regulated at 12 and 24 hpi with additional respect to distance from infection location (Mulema and Denby, 2012). Rezzonico et al. (2017) found for the tomato-*B. cinerea* pathosystem differentially gene regulation not only between mock- and pathogen-inoculated samples but also between different pathogens and inoculation methods. Recently, in barley-powdery mildew interaction specific reflectance bands were shown to correlate to pathogen-induced gene expression changes (Kuska et al., 2019). The genes *JIP23* (jasmonate induced proteins), *RuBisCO* (ribulose-1,5-bisphosphate carboxylase small subunit), and *PR5* (encoding a thaumatin-like protein) among seven tested genes were analyzed by means of quantitative real time PCR in susceptible barley variety *Hordeum vulgare* infected with *Blumeria graminis* f.sp. *hordei*. Their expression in five sampling times during 72 hpi showed high relevance in local neighborhood analysis and correlated with reflectance from diseased tissue strongest in the wave bands from 660 to 820 nm (Kuska et al., 2019).

Summarizing, our work demonstrated “off-the-shelf” multispectral cameras to be suitable for early, pre-symptomatic detection of gray mold infections in tomato leaves. Computational post processing to correct multiple lenses systems-derived shifts are complicated, error prone and time consuming. In future experiments single-lens systems should be favored. Future experiments should investigate the process adaptations toward field conditions and multiple environmental influences such as leaf angle and artifacts due to non-homogeneous background, disease severity, biotic and abiotic stresses.

## Author Contributions

JF designed the experiments. FR conducted all experiments, set up multispectral imaging, and collected imagery. MG extracted the data from imagery. BP conducted the statistical analyses. JF, MG, and BP wrote the manuscript. All authors have read and approved the final version of the manuscript.

## Conflict of Interest Statement

The authors declare that the research was conducted in the absence of any commercial or financial relationships that could be construed as a potential conflict of interest.

## Footnotes

1 e.g., http://ephytia.inra.fr

2 https://www.psm.admin.ch/de/schaderreger/10373 (accessed April 2019).

3 www.fao.org/faostat/en/ (accessed September 2016).

4 accessed 18.10.2018
